# Intakes of plant foods, fibre and fat and risk of breast cancer – a prospective study in the Malmö Diet and Cancer cohort

**DOI:** 10.1038/sj.bjc.6601516

**Published:** 2004-01-06

**Authors:** I Mattisson, E Wirfält, U Johansson, B Gullberg, H Olsson, G Berglund

**Affiliations:** 1 Department of Medicine, Surgery and Orthopaedics, Lund University, Malmö University Hospital, SE-205 02 Malmö, Sweden; 2 Department of Community Medicine, Lund University, Malmö University Hospital, Entrance 59, SE-205 02 Malmö, Sweden; 3 Department of Oncology, Lund University, SE-221 85 Lund, Sweden

**Keywords:** fat, fibre, plant foods, postmenopausal breast cancer, prospective study

## Abstract

The objective of this study was to investigate prospectively the associations between intakes of plant foods, fibre and relative fat and risk of breast cancer in a subsample of 11 726 postmenopausal women in the Malmö Diet and Cancer cohort. Data were obtained by an interview-based diet history method, a structured questionnaire, anthropometrical measurements and national and regional cancer registries. During 89 602 person-years of follow-up, 342 incident cases were documented. Cox regression analysis examined breast cancer risks adjusted for potential confounders. High fibre intakes were associated with a lower risk of postmenopausal breast cancer, incidence rate ratio=0.58, 95% CI: 0.40, 0.84, for the highest quintile of fibre intake compared to the lowest quintile. The combination high fibre–low fat had the lowest risk when examining the effect in each cell of cross-classified tertiles of fibre and fat intakes. An interaction (*P*=0.049) was found between fibre- and fat-tertiles. There was no significant association between breast cancer risk and intakes of any of the plant food subgroups. These findings support the hypothesis that a dietary pattern characterised by high fibre and low fat intakes is associated with a lower risk of postmenopausal breast cancer.

Breast cancer is the most common cancer in women worldwide, but incidence rates vary greatly between countries (World Cancer Research Fund, 1997). Several biological processes associated with diet might be relevant to breast cancer aetiology. Much of the previous research on breast cancer and diet has focused on the total fat intake and disease risk, but results from different studies are conflicting (Lee and Lin, 2000; Willett, 2001). Plant foods have attracted less interest, although they are sources of several compounds that might be of importance in breast cancer development. These compounds include vitamins (e.g., ascorbic acid, folic acid, vitamin E and *β*-carotene), fibre and various phytochemicals (flavonoids, phyto-oestrogens, indols, carotenoids, etc.). Phyto-oestrogens especially have the potential to influence breast cancer risk. They are similar in structure to oestrogen and may compete with endogenous oestrogen for the oestrogen receptors (Cassidy *et al*, 2000).

High fat consumers in the Malmö Diet and Cancer (MDC) cohort have substantially lower intakes of fruit, vegetables and whole grain bread compared to low fat consumers and intakes of antioxidants and fibre are inversely associated with the relative fat intake (Mattisson *et al*, 2003a). Thus, it is of interest to examine if the association between plant food intake and breast cancer risk is influenced by fat intake. The aims of this study are to examine (1) if plant foods and fibre intakes are associated with breast cancer risk, and (2) if these associations are influenced by fat intake. In addition, the combined effect of fat and fibre intakes on breast cancer incidence was examined by cross-classifying energy-adjusted tertiles of fat and fibre.

## MATERIAL AND METHODS

### Malmö Diet and Cancer

The MDC study is a prospective cohort study in Malmö, a city in the south of Sweden with approximately 250 000 inhabitants. The MDC source population was, in 1991, defined as all persons living in the City of Malmö, born between 1926 and 1945. However, in May 1995, the cohort was extended to also include all women born between 1923 and 1925 and 1946 and 1950, and all men born between 1923 and 1925. With this extension, 74 138 persons constitute the source population. Both personal invitation letters and passive recruitment strategies (e.g., advertisement in newspapers and on buses) directed towards the community were used to recruit study participants. During the first 2 years, approximately 50% of the subjects were recruited by passive recruitment. However, during 1994–1996, less than 5% were passively recruited. Inadequate Swedish language skills and mental incapacity were the only exclusion criteria. When the baseline examinations closed in October 1996, 28 098 participants had completed all parts. More details of recruitment and the cohort are described elsewhere (Manjer *et al*, 2002). The ethical committee at Lund University has approved the MDC (LU 51–90).

The participants visited the MDC screening centre twice. During the first visit, groups of 6–8 participants were informed about the study, instructed how to register meals in the menu-book and how to fill out the diet questionnaire and the extensive questionnaire covering socio-economic factors and lifestyle. Project nurses took blood samples, blood pressure and anthropometric measurements. Participants completed all questionnaires at home. The dietary interview was conducted and the socio-economic questionnaire checked at the second visit, approximately 10 days after the first.

### Study population

In total, 11 726 postmenopausal women were included in this subsample from the MDCS cohort. This study used an age-based definition of menopausal status (Morabia and Flandre, 1992). Eligible participants for this analysis were women who were 50 years or older at baseline examination. All prevalent cancer cases, except cervix cancer *in situ* and squamous cell carcinoma of the skin, were excluded. Women born between 1923 and 1925 and 1946 have shorter follow-up, due to the MDC study design.

### Case definition and ascertainment

The National Swedish Cancer Registry provided data until December 1999; additional information until end of follow-up (31 December 2001) was obtained from the Southern Swedish Regional Tumour Registry. Cases were women diagnosed during follow-up with invasive breast cancer or breast cancer *in situ*. Information on vital status was obtained from the National Tax Board that provides up-to-date information on vital status for all Swedish residents. Cases contributed person-time from date of enrolment until time of diagnosis. Noncases contributed person-time from the date of enrolment until death (470 women), migrating from Sweden (57 women) or end of follow-up (31 December 2001), whichever was the first. In total, 312 invasive and 30 *in situ* incident breast cancer cases were documented during 89 602 years of follow-up.

### Dietary data

The MDC method is an interview-based, modified diet history method. Briefly, it combines a 7-day menu book for recording of cooked meals, beverages, drugs, natural remedies and nutrient supplements and a diet questionnaire for assessment of meal pattern, consumption frequencies and portion sizes of regularly eaten foods. The mean daily intakes of food were calculated and converted to energy and nutrient intakes using PCKost2-93 from the National Food Administration in Uppsala, Sweden. The MDC method is described in detail elsewhere (Mattisson *et al*, 2001; Wirfält *et al*, 2002b). The relative validity of the MDC method was evaluated in a sample of Malmö residents, 105 women and 101 men, 50–69 years old, using 3 days of weighed records every other month during a year, as a reference method. The energy-adjusted Pearson correlation coefficients, between the reference method and the MDC method administered after the 12-month reference period, were 0.69 for both fibre and fat intake, (Riboli *et al*, 1997), 0.53 for vegetables, 0.77 for fruit, 0.51 for potatoes, 0.58 for bread and 0.24 for rice and pasta (Elmståhl *et al*, 1996).

### Variables

This study examined gram amounts of fibre and of the following 16 plant food-group variables: total fruit (including berries), fruit juice, vegetables, total fruit and vegetables, total fruit, vegetables and fruit juice, potatoes boiled, potatoes fried and deep fried, high-fibre bread, low-fibre bread, total bread, high-fibre cereals, low-fibre cereals, buns and cookies, nuts, snacks and rice and pasta.

Total fat, n-6 polyunsaturated fatty acids (PUFAs), wine and fermented milk were also examined as potential confounders because these variables have been associated with breast cancer risk in previous studies in the MDC cohort (Wirfält *et al*, 2002a; Mattisson *et al*, 2003b; Wirfält *et al*, 2003).

Energy-adjusted variables of total fat, n-6 PUFAs, fibre, total fruit and vegetables and fermented milk variables were defined as the residuals obtained when regressing the specific nutrient or food group on total energy intake (EI). Five exposure categories were created based on the quintile ranking of participants on residuals. In addition, for fat and fibre, a three-category variable was defined, based on the tertile ranking of participants on residuals. Information on wine consumption (cl day^−1^) was converted into a four-category variable based on the ranking of absolute intakes: wine-abstainers (zero consumption in menu book and no wine consumption during the last year indicated in the questionnaire); low consumers (wine intakes below or equal to the median intake); medium consumers (wine intakes above median intake but below or equal to the 97.5th percentile; high consumers (wine intakes above the 97.5th percentile).

In September 1994, the dietary data processing procedure was slightly altered (Wirfält *et al*, 2002b). *Method version* (indicating data collection before or after 1 September 1994), *diet interviewer* and *season of diet interview* were used as covariates to control for undue variation in the dietary data collection over time.

*Age at baseline* was obtained from the 10-digit person identification number.

Information on reproduction, socio-economic and life style factors was collected by a structured multiple-choice questionnaire.

*Past change of dietary habits* was based on the question ‘have you substantially changed your dietary habits because of illness or another reason?’

*Age at menarche* was used as a continuous variable. *Age at the birth of the first child* was divided into four categories (⩽24 years, 24–30 years, >30 years and ‘no children’); missing information (2.0%) was recoded to ‘no children’.

*Current use of hormone therapy* was based on the question ‘which medicines do you use on a regular basis?’ in combination with the 7-day recording of drug use in the menu book, and used as a dichotomous (yes/no) variable (Merlo *et al*, 2000).

Participants were divided into four categories according to their *highest level of education*: ⩽8 years, 9–10 years, 11–12 years and college education/university degree.

Participants indicated the number of minutes per week, separately for the four seasons, for 17 different physical activities (Haftenberger *et al*, 2002). The question was adapted from the Minnesota Leisure Time Physical Activity Questionnaire (Taylor *et al*, 1978; Richardsson *et al*, 1994). The number of minutes of each activity was multiplied with an activity coefficient and an overall *leisure time physical activity score* was created. The score was divided into quintiles and further categorised as low (quintile 1), moderate (quintile 2–4) or high (quintile 5).

Standing *height* was measured with a fixed stadiometer calibrated in centimetres. *Weight* was measured to the nearest 0.1 kg using a balance-beam scale with subjects wearing light clothing and no shoes. *Waist* was measured midway between the lowest rib margin and iliac crest. Owing to the high correlation (*r*=0.86) between BMI and waist circumference, we decided to use only one obesity measure as a covariate. Waist was chosen because central obesity rather than general obesity has been suggested to predispose an individual to the development of breast cancer (Stoll, 1996).

To evaluate reported EIs, the ratio between reported total EI and basal metabolic rate (BMR) was calculated. Basal metabolic rate was estimated using the equation recommended by WHO; based on age, sex, weight and height (FAO/WHO/UNU, 1985).

### Statistical methods

SPSS statistical computer package (version 10.0; SPSS Inc., Chicago, IL, USA) was used for the statistical analyses. Crude median and interquartile intakes of food groups and fibre were calculated for cases and noncases. All dietary variables were log-transformed to reduce skewness. Before transformation, a very small amount (=0.01) was added to food intake variables in order to handle zero consumption. *T*-test examined differences in the mean food and fibre intakes between cases and noncases.

The multivariate associations between food intake and breast cancer risk were examined with Cox regression models. Firstly, the influence of each of the 16 plant food-groups and dietary fibre on breast cancer risk was examined as continuous variables, while adjusting for diet interviewer, season of diet interview, method version, age at baseline, past food habit change and total energy (basic model). Food groups were further examined, in ‘full models’ also including established and potential risk factors (i.e., height, waist, current hormone use, age at the birth of the first child, age at menarche, leisure time physical activity and educational level). Secondly, the risk across quintiles of total fruits and vegetables and dietary fibre was estimated with both the ‘basic’ and the ‘full’ models. The ‘full models’ were extended to include adjustment for energy-adjusted quintiles of total fat, n-6 PUFAs, fermented milk or wine intake categories since these have been found to influence breast cancer risk in the MDC cohort. The models were also repeated while excluding women less than 51 years at baseline (*n*=719, including 27 cases); women diagnosed with incident breast cancer within 1 year from baseline examination (31 cases) or *in situ* cancer cases (30 cases).

Finally, energy-adjusted tertiles of fat and fibre intakes were cross-classified, resulting in a nine-category grid. Cox regression estimated the breast cancer risk across all nine categories, with the combination high fibre–low fat as the reference category, since this category was believed to represent the lowest breast cancer risk (World Cancer Research Fund/American Institute for Cancer Research, 1997). This model was also extended to include adjustment for n-6 PUFA quintiles, wine intake categories and fermented milk quintiles. Further, we tested for interaction between tertiles of fibre and fat.

## RESULTS

Table 1

**Table 1:** Intakes of plant foods and fibre at baseline examination in cases and noncases in the Malmö Diet and Cancer cohort

	**Cases (*n*=342)**		**Noncases (*n*=11 384)**	
	**Median (g day^−1^)**	**IQ range**	**Median (g day^−1^)**	**IQ range**
Total fruits and berries	197	148	196	154
Total fruit juices	0.78	143	0.64	107
Total vegetables	154	111	158	112
Total fruits, berries and total vegetables	370	184	369	200
Total fruits and berries, total vegetables and juices	444	234	430	246
Boiled potatoes	76	58	76	61
Fried and deep fried potatoes	0	20	0	20
High-fibre bread	34	43	34	39
Low-fibre bread	41	50	40	49
Total bread	84	55	82	56
High-fibre cereals	37	42	37	42
Low-fibre cereals	60	56	61	52
Buns and cookies	32	39	32	36
Rice/pasta	7	15	8	15
Nuts	0	1.2	0	1.1
Snacks	0	0.1	0	0.5
Fibre	18.2	7.8	18.4	7.8

shows the median intakes of the different plant food-groups and fibre in cases and noncases. There were no significant differences in the mean intakes of food groups and fibre between cases and noncases (data not shown). The baseline characteristics of cases and noncases are described in detail elsewhere (Mattisson *et al*, 2003b). In short, cases were younger at baseline (a consequence of the MDC study design and the shorter follow-up among many older women), taller, more often current users of hormone therapy and had more often medium–high physical activity.

In the multivariate analysis, there were no significant associations between food group intakes (continuous) and breast cancer risk (data not shown). Fibre intake (continuous) was significantly associated with decreased risk, both in the ‘basic model’ (*P*=0.044) and in the ‘full model’ (*P*=0.012).

We also examined the associations between energy-adjusted quintiles of fibre and of fruit and vegetables and breast cancer risk. The highest quintile of fibre intake was associated with a lower breast cancer risk both in the basic and full models (Table 2

**Table 2:** Incidence rate ratios of postmenopausal breast cancer according to energy-adjusted quintiles of intakes of fibre and ‘total fruit and vegetables’ in the Malmö Diet and Cancer cohort 1991–2001

	**Basic model^a^**			**Full model^b^**			**Adjusted for total fat**	
	**Person-years/cases**	**IRR**	**95% CI**	**Person-years/cases**	**IRR**	**95% CI**	**IRR**	**95% CI**
*Fibre quintiles (intake*^c^)								
1 (12.5 g)	17 026/75	1		16 429/73	1		1	
2 (16.0 g)	17 531/61	0.78	0.55, 1.09	17 052/59	0.75	0.53, 1.05	0.76	0.54, 1.08
3 (18.3 g)	17 840/64	0.78	0.56, 1.10	17 373/63	0.74	0.52, 1.04	0.78	0.55, 1.12
4 (21.0 g)	18 232/82	0.93	0.68, 1.28	17 766/78	0.86	0.62, 1.19	0.95	0.67, 1.35
5 (25.9 g)	18 828/60	0.64	0.45, 0.91	18 305/56	0.58	0.40, 0.84	0.66	0.44, 0.99
			*P* for trend=0.120			*P* for trend=0.056		*P* for trend=0.399

*Total fruit and vegetables quintiles (intake*^c^)								
1 (210 g)	17 420/64	1		16 902/61	1		1	
2 (297 g)	17 801/80	1.21	0.87, 1.68	17 203/77	1.20	0.86, 1.69	1.24	0.88, 1.73
3 (370 g)	18 010/63	0.92	0.65, 1.38	17 513/60	0.91	0.63, 1.30	0.96	0.67, 1.38
4 (453 g)	18 041/77	1.12	0.80, 1.56	17 600/77	1.13	0.81, 1.59	1.21	0.85, 1.71
5 (600 g)	18 185/58	0.82	0.57, 1.17	17 710/54	0.78	0.54, 1.13	0.86	0.58, 1.25
			*P* for trend=0.293			*P* for trend=0.275		*P* for trend=0.652

a Adjusted for diet interviewer, season of diet interview, method version, age, change of dietary habits.

b Adjusted for diet interviewer, season of diet interview, method version, age, change of dietary habits, total energy, current hormone use, age at first child, height, waist, leisure time physical activity, age at menarche, educational level.

c Crude median intakes of fibre or fruit and vegetables.

); however, the trend across quintiles was not significant. Adjusting for wine, n-6 PUFAs or fermented milk did not influence the association between fibre and breast cancer risk (data not shown). Adjusting for total fat attenuated the association but the lower risk in the fifth fibre quintile remained significant (Table 2). None of the quintiles of the total fruit and vegetables variable was significantly associated with breast cancer risk (Table 2).

Excluding women less than 51 years at baseline, women diagnosed with incident breast cancer within 1 year from baseline examination or cases with *in situ* breast cancer did not change risk estimates for high fibre intakes. However, the trend across fibre quintiles became significant when we excluded *in situ* cases or women below 51 years of age at baseline.

Table 3

**Table 3:** Incidence rate ratios^a^ of postmenopausal breast cancer according to combined fibre and fat intake tertiles in the Malmö Diet and Cancer cohort 1991–2001

	**Fat tertiles (E%^b^)**								
	**1 (32.0)**			**2 (37.5)**			**3 (42.9)**		
**Fibre tertiles (fiber intake^c^)**	**IRR**	**95% CI**	**Person-years/cases**	**IRR**	**95% CI**	**Person-years/cases**	**IRR**	**95% CI**	**Person-years/cases**
1 (13.7)	1.50	0.82, 2.73	3868/14	1.01	0.60, 1.71	8058/20	1.88	1.29, 2.73	15 793/72
2 (18.3)	1.26	0.79, 2.02	8117/27	1.78	1.21, 2.63	11 841/56	1.35	0.86, 2.11	9061/32
3 (23.8)	1		17 561/50	1.56	1.02, 2.37	9090/39	1.99	1.17, 3.38	3538/19

a Adjusted for diet interviewer, season of diet interview, method version, age, change of dietary habits, total energy, current hormone use, age at first child, height, waist, leisure time physical activity, age at menarche and educational level.

b Crude median energy percentage of fat (%).

c Crude median intakes of fibre (g day^−1^).

shows the breast cancer risk in each cell of cross-classified tertiles of fibre and fat intakes. Compared with the group ‘high fibre–low fat’, all other combinations had elevated estimates. Adjusting for quintiles of energy-adjusted n-6 PUFAs and fermented milk or wine intake categories only marginally influenced outcome, and the risk estimates became slightly lower (data not shown). The test for interaction between tertiles of fibre and fat was significant, *P*=0.049.

## DISCUSSION

We found that high fibre intakes, quintile five compared with quintile one, was associated with a significant 40% reduction in breast cancer risk. The median fibre intake in quintile five was 25.9 g day^−1^, which corresponds to the lower limit of the recommended daily fibre intake in the Nordic countries (Nordiska ministerrådet, 1996). The lower risk associated with high fibre intakes was independent of total fat, n-6 PUFAs, fermented milk and wine intakes. When we examined the combined effect of fibre and fat by cross-classifying tertiles of fibre and fat, the combination high fibre–low fat had the lowest risk of breast cancer. There were no significant associations between intakes of any of the plant food subgroups and breast cancer risk. *Ad hoc* analyses showed that the protective effect of high fibre intakes remained when we adjusted for intakes of antioxidants (ascorbic acid, *β*-carotene, selenium, *α*-tocopherol and folate; data not shown).

The results from epidemiological studies are compatible both with a protective effect, and with no effect, of fibre on breast cancer risk. For instance, in a case–control study in China, women in the lowest tertile of crude fibre intake and highest tertile of fat intake had a 2.9-fold increased risk for breast cancer relative to those in the highest tertile of crude fibre intake and lowest tertile of fat intake (Yuan *et al*, 1995). Howe *et al* (1990) found a significant (*P*=0.002) protective effect of fibre in a combined analysis of 12 case–control studies, but this effect was not independent of *β*-carotene and vitamin C. No association was found in the Nurse's Health Study (Willett *et al*, 1992). A Canadian prospective study found a significant reduced risk in the highest fibre quintile relative to the lowest quintile (Rohan *et al*, 1993).

There are several hypotheses on the influence of fibre on breast cancer risk. Fibre might modulate the enterohepatic recirculation of oestrogens leading to increased faecal excretion of oestrogens and reduced levels of circulating bioavailable oestrogen (Gerber, 1998). Breast cancer has been suggested to be associated with the insulin resistance syndrome (Kaaks, 1996; Stoll, 1999); a fibre-rich diet could slow digestion and absorption of carbohydrates and influence plasma insulin response (Slavin, 2000). Minor constituents, like antioxidants, phytate and especially phyto-oestrogens, present in fibre-rich foods, might be biologically active components (Gerber, 1998; Cohen, 1999). Phyto-oestrogens (plant food components like isoflavones, lignans and stilbenes) might act both as partial oestrogen agonists and as antagonists, which makes it difficult to predict their effect (Cassidy *et al*, 2000). Lignans are present in most fibre-rich foods and they might be important sources of phyto-oestrogens, especially in Northern European diets. Epidemiological studies on phyto-oestrogens and breast cancer are scarce and prospective studies have until now shown nonsignificant results (Peeters *et al*, 2003).

We cross-classified tertiles of fibre and fat in order to illustrate the influence of different fibre and fat intake combinations on breast cancer risk. Risk estimates in groups with low fibre and high fat intakes were lower than expected as indicated by the significant test for interaction between fibre and fat tertiles (i.e., not a multiplicative effect). Although the combination high fibre–low fat had the lowest risk, there is no obvious pattern in breast cancer risk across the nine categories, which implies that other factors also might influence the observed risk estimates. There was no large difference in n-6/n-3 ratio across the nine categories. It varied from 4.99 in the medium fibre–high fat group to 5.25 in the medium fibre–low fat group. However, wine intakes varied substantially across the nine groups. There was a tendency of lower prevalence of high wine intakes with both increasing fibre intakes and increasing fat intakes. The prevalence of high wine intakes was 7.2% in the low fibre–low fat group and 0.0% in the high fibre–high fat group. Fermented milk intakes tended to decrease with higher fat intakes and increase with higher fibre intakes. The highest mean intake was found in the medium fibre–low fat group and the lowest mean intake in the medium fibre–high fat group. This is an interesting finding since it is possible that fermented milk influences the gut flora, which could lead to an increased activation of dietary lignans to active mammalian lignans (Cassidy *et al*, 2000; Parr and Bolwell, 2000; Adlercreutz, 2002). In fact, a case–control study found a synergistic protective effect of fibre and fermented milk on breast cancer risk. The study concluded that a dietary pattern combining low fat, high fibre and fermented milk might provide substantial protection against breast cancer (van't Veer *et al*, 1991). Although the extended model was adjusted for these nutrients, residual confounding might remain. It is also plausible that intakes of other nutrients and foods vary across the nine groups and contributes to the risk estimates observed. In addition, the numbers of person-years and cases within each of the nine categories influences the stability of risk estimates. The most stable estimates are found in the high fibre–low fat, medium fibre–medium fat and low fibre–high fat groups.

In this study, we found no association between total fruit and vegetable intakes and breast cancer risk. The intakes of fruit and vegetables in the MDC cohort might be too low and too homogenous to examine any effect. As indicated by food intake data from the calibration study within the European Prospective Investigation into Nutrition and Cancer (EPIC), the MDC subcohort has among the lowest intakes of both fruit and vegetables in the EPIC study centres (Agudo *et al*, 2003).

Although epidemiological data support the apparent inverse association between consumption of fruits and vegetables and breast cancer risk, studies are not consistent overall (World Cancer Research Fund, 1997). For instance, a meta-analysis comprising published data from 26 cohort and case–control studies found a negative association between vegetables and breast cancer risk, but no association between fruit and breast cancer risk (Gandini *et al*, 2000). A pooled analysis of eight prospective cohort studies found no significant association between vegetable intakes, fruit intakes and risk of breast cancer (Smith-Warner *et al*, 2001). The amount eaten and the specific choice of fruit and vegetable in the different cohorts might be some of the explanations behind inconsistent results.

During the initial years of recruitment, over 50% of the participants were recruited by community-directed activities (Manjer *et al*, 2002). It is plausible that these ‘volunteers’ were more health conscious and had a more salutatory behaviour compared to those who joined after a personal invitation. The differences seen in the numbers of person-years in the different fibre quintiles might be explained by higher energy-adjusted fibre intakes in women who joined early.

Under-reporting of energy is a major concern when using self-reported dietary data (Black *et al*, 1991; Black, 2000). In this study, we used energy-adjusted variables, thus trying to reduce the effect of under-reporting. There was no significant difference in EI/BMR ratios across quintiles of fibre or total fruit and vegetables (data not shown). There were small differences across cross-classified fibre and fat tertiles but no obvious pattern; the EI/BMR ratio varied from 1.41 in the high fibre–high fat category to 1.48 in the high fibre–medium fat category. Although an effect never can be excluded, it is not likely that under-reporting may have a major impact on the outcomes of this study.

To conclude, a dietary pattern characterised by high fibre and low fat intakes was associated with a lower risk of postmenopausal breast cancer. The low intake levels of fruit and vegetables in the MDC cohort may contribute to the absence of an association between breast cancer risk and intakes of fruit and vegetables in this study.
